# From clinician to coach: promoting early relational health and language development in primary pediatric care

**DOI:** 10.3389/fped.2026.1900464

**Published:** 2026-07-16

**Authors:** Claire Samuel, Sarah Behrens, Brenda Salley

**Affiliations:** 1 University of Kansas School of Medicine, Kansas City, KS, United States; 2 Department of Pediatrics, University of Kansas Medical Center, Kansas City, KS, United States

**Keywords:** clinician-delivered-coaching, collaborative care, early relational health, language development, patient engagement, pediatric primary care, physician communication, well-child visits

## Abstract

Coaching is a promising approach to professional development that emphasizes collaboration, goal setting, and skill building through a supportive, non-competitive relationship. Grounded in an individualized approach, coaching engages individuals in defining and pursuing goals that promote professional growth and positive health behavior change. While existing literature largely positions clinicians as recipients of coaching, far less attention has been given to how physicians might apply these principles in direct interactions with patients and families. This perspective explores how clinician-delivered coaching in pediatric primary care - particularly during well-child visits - can strengthen early relational health and language development. Core coaching competencies including trust-building, collaborative goal setting, reflective dialogue, and strengths-based feedback, offer a framework for reimagining the clinician's role as a coach. Integrating these strategies into routine care has the potential to enhance parent engagement, reinforce family-centered practice, and extend the preventative impact of primary care. Evidence from physician-led coaching in professional development and peer mentoring suggests that physicians can successfully adopt coaching roles, supporting the application of these principles within pediatric primary care.

## 1 Introduction

Coaching has emerged as an effective approach to professional development in medicine, supporting clinician learning, skill acquisition, and sustained performance. In this context, coaching is conceptualized as both a communication approach and a clinical role grounded in learned competencies that physicians intentionally develop and apply to support professional growth and facilitate patient and family behavior change. However, its application has largely been limited to physicians as recipients, with far less attention to how these same principles might inform physicians' interactions with patients and families. Reframing physicians as relational coaches within clinical care represents an opportunity to extend the impact of coaching beyond professional development and into patient-centered practice. This perspective provides a framework for how clinician-delivered coaching (e.g., collaborative goal setting, strengths-based feedback) within pediatric primary care may strengthen parent self-efficacy, support responsive caregiver-child interaction, advance early relational health, and optimize child developmental outcomes.

Effective continuous professional development is fundamental for maintaining high-quality care. Yet studies suggest that as physicians move further from formal training, clinical performance may decline, reflecting challenges in sustaining competence over time (1). Traditional models of continuing education are often passive, difficult to individualize, and limited in their ability to meaningfully change practice (1). In contrast, achieving and maintaining expertise requires deliberate practice, including repeated skills application, self-reflection, and specific, observation-based feedback (2). Following residency, however, physicians have fewer opportunities for structured observation and feedback from trusted colleagues, contributing to gaps in ongoing performance development. Coaching has emerged as a strategy to address this gap (2).

Importantly, the same challenges that coaching addresses for physicians – translating knowledge into sustained behavior change – are also experienced by families navigating early child development. Core coaching principles, including collaborative goal setting, reflective dialogue, feedback, and skill practice, are therefore highly applicable to parent-child interactions. Integrating these strategies into pediatric primary care reframes the physician's role to include functioning as a coach alongside their traditional clinical responsibilities. This application is conceptual and proposes that core coaching competencies may be transferable across professional and caregiver-facing contexts. However, the extent to which these competencies translate directly, or require adaptation to meet the needs of families within pediatric care, has not yet been empirically established. Competencies such as observation, strengths-based feedback, collaborative goal setting, and reflective dialogue are likely to transfer across settings, whereas components of the coaching implementation strategy (e.g., visit structure, dose, follow-up, integration within family-centered workflows) will likely require adaptation for time-limited pediatric primary care encounters. This approach has the potential to enhance parent engagement, strengthen early developmental relationships, and align with family-centered models of care.

## 2 Coaching across contexts in medicine

Physicians have increasingly engaged in coaching roles across professional and clinical settings, including professional development, faculty coaching, and peer coaching among practicing physicians. Across these contexts, structured coaching interventions have been associated with improvements in communication, professional growth, and clinical performance, while cultivating trust and accountability. The concept of coaching has been applied across multiple contexts, including physician coaching in medical education, health coaching, and parent coaching. Although these traditions have developed independently, they share core principles of collaborative partnership, goal-directed learning, and behavior change. This perspective integrates these complementary coaching traditions to examine how clinician-delivered coaching can be applied within pediatric primary care. These findings support the feasibility of physicians functioning as coaches in medical settings and provide a foundation for considering how coaching principles may be adapted to patient and family interactions. Pediatric primary care, particularly during well-child visits, offers a unique, relationship-based context in which coaching-oriented interactions can be applied. The following sections provide an overview of how coaching has been used across domains and how these models inform its application in pediatric care.

### 2.1 Coaching in skill acquisition

Coaching models are widely applied to support the acquisition of technical and clinical skills, often through “train-the-trainer” approaches (3). These models emphasize structured observation, deliberate practice, goal-setting, and timely feedback to promote skill mastery. For example, a remote coaching model designed to enhance clinician capacity in wheelchair skills training combined self-directed learning, guided practice, and asynchronous expert feedback (3). This approach improved clinician confidence and skills transmission, illustrating how coaching can build both competence and teaching capacity. Such models are directly applicable to pediatric primary care, where clinicians support parents in learning and applying new skills related to child development.

### 2.2 Coaching in physician professional development

In professional development, coaching supports a shift from passive continuing education toward active, practice-based learning (1). A central component is observation (direct or indirect), which allows for identification of strengths and opportunities for growth in real-world settings. Coaches collaborate with physicians to set individualized goals and guide change across both the size of change (from refining existing practices to adopting new skills) and the level of change (from individual behaviors to broader practice systems) (1). This model demonstrates how coaching facilitates sustained behavior change, a principle that is equally relevant when supporting families in clinical care.

### 2.3 Coaching in patient behavior change and family contexts

Health coaching is a collaborative, patient-centered approach that empowers individuals to manage health behaviors and make informed decisions. Through goal setting, action planning, and ongoing support, coaching has been associated with improved management of chronic diseases such as asthma, diabetes, and hypertension, while offering a potentially cost-efficient model of care (4). Central to this approach is a trusting relationship that fosters self-efficacy, reflection, and active engagement.

While traditionally applied to chronic disease management, these principles are highly relevant to early relational health and parenting practices. Supporting parents in areas of responsive caregiving, communication, and stress regulation requires not only information, but also sustained behavior change, mirroring the challenges addressed through coaching in other contexts. Parent coaching models, including those grounded in strengths-based and positive psychology frameworks, emphasize collaboration, non-judgement, and the co-creation of goals and strategies (5). In these approaches the clinician or coach partners with parents to build on existing strengths, navigate everyday challenges, and promote healthy parent-child interactions.

Integrating this approach into pediatric primary care can create an opportunity to move beyond education alone and toward relational, skill-based support that may strengthen early developmental outcomes. In this way, coaching serves as a unifying framework that links physician development, patient behavior change, and early relational health within routine care.

Importantly, the evidence base supporting coaching spans multiple related but distinct domains. Strong evidence exists for coaching in physician professional development, with growing evidence in health coaching for patient behavior change and parent-mediated interventions. In contrast, direct evidence for clinician-delivered coaching within pediatric primary care remains limited. This perspective draws on converging evidence across these domains to inform its application in well-child care.

Although coaching shares characteristics with several established approaches used in healthcare, it is conceptually distinct in its purpose and application (Table 1).

**Table 1:** Coaching and related approaches.

Approach	Definition
Coaching	A collaborative, goal-oriented approach that supports learning, skill development, self-efficacy, and sustained behavior change through guided reflection and partnership.
Mentorship	A developmental relationship in which a more experienced individual provides guidance, advice, and career or personal support (6).
Health Education	A teaching-focused approach that emphasizes the transfer of health information and knowledge to patients or families.
Counseling	A process focusing on psychological or emotional challenges, past experiences, and mental health diagnoses to facilitate emotional regulation, healing, and personal understanding.
Motivational Interviewing	A specific evidence-based communication technique designed to strengthen an individual's intrinsic motivation for behavior change (7).
Parent-mediated Intervention	An intervention in which caregivers are taught strategies to implement with their child to improve developmental or behavioral outcomes.
Clinical Supervision	An evaluative process that provides oversight, competency assessment, and support to ensure safe and effective clinical practice.

## 3 The role of coaching in primary pediatric care

Emerging evidence suggests that coaching can be feasibly integrated into busy pediatric primary care settings and is valued by both clinicians and families (2). Within well-child visits, coaching can be incorporated through brief, targeted interactions embedded into routine care, with intensity tailored to family needs and time constraints. Opportunities for follow-up reflection, and goal review can occur longitudinally across visits, aligning with the ongoing nature of pediatric care.

Although the specific implementation of coaching will vary across clinical settings and family needs, Table 2 provides an illustrative example of how core coaching competencies may be integrated into a routine well-child visit.

**Table 2:** Example framework for integrating coaching into a well-child visit.

Coaching Competencies	Example During Well-Child Visit
Observation	Observe caregiver-child interactions during play, conversation, or reading to identify existing strengths and areas for support.
Strengths based feedback	Affirm existing strengths to reinforce positive behaviors.
Asking Permission	Ask whether the caregiver is open to discussing an observation or hearing a suggestion before providing recommendations.
Collaborative Goal Setting	Work collaboratively to identify one goal that aligns with the family's priorities and daily routines.
Skill Development	Suggest or model a developmentally supportive strategy.
Follow-Up	Follow up on the caregiver's experience, celebrate successes, discuss barriers, and refine goals as needed to support continued growth.

In practice, adopting a coaching approach shifts the clinical encounter from primarily directive guidance or education toward collaborative problem solving. Pilot studies demonstrate that clinicians using coaching strategies are more likely to ask permission before offering guidance (e.g., “Would you like to hear an idea that could help?”), elicit parental perspectives (“What feels most important to focus on right now?”), and support families in translating recommendations into realistic daily routines (“What is one thing you’d like to try this week?”) (2, 8). These shifts promote parent reflection, skill development, and relational engagement.

Parents report high acceptability of this approach, valuing the emphasis on partnership and empowerment. Coaching within well-child visits allows clinicians to bridge developmental knowledge with relational support, moving beyond information delivery toward a model in which parents are supported as active participants in their child's development. In this way, the pediatrician functions not only as a medical expert, but also as a collaborative partner who supports goal setting, contextualized decision-making, and growing parental confidence.

## 4 Early relational health and coaching in pediatric primary care

The American Academy of Pediatrics (AAP) has identified early relational health as a foundational component of lifelong health and development, emphasizing that the caregiver–child relationship are as critical to preventive care as immunizations and developmental screening. Early relational health refers to the quality of early caregiver-child relationships that support social, emotional, and cognitive development through responsive, nurturing, and consistent engagement. Because pediatric clinicians maintain longitudinal relationships with families through routine well-child visits, primary care offers a natural setting to promote early relational health at scale.

Coaching may offer a practical mechanism for operationalizing early relational health within clinical care. The core practices of coaching - observation, affirmation, modeling, and guided reflection – directly mirror the behaviors that strengthen caregiver-child relationships. In this way, coaching is not separate from early relational health, but an inferred mechanism for embedding early relational health principles into everyday pediatric encounters. By emphasizing collaboration, strengths-based guidance, and goal setting, coaching enables clinicians to translate developmental science into actionable strategies that families can apply in real time.

Several established interventions illustrate how coaching can be integrated into pediatric primary care to support early relational health. Many AAP-endorsed ERH programs leverage well-child visits as universal touchpoints to promote responsive caregiving and language-rich interaction (9). For example, programs such as **Reach Out and Read** (10) leverage routine well-child visits to promote shared reading and language rich interactions, with clinicians guiding parents to read aloud, engage in conversation, and share stories during routine visits. In doing so, they support the development of consistent language rich habits and strengthen early literacy. Similarly, **PlayReadVIP** incorporates individualized coaching strategies, such as video-based feedback and strengths-based reflection, to enhance caregiver awareness, confidence, and responsiveness (11).

Additional interventions, including **TREE (Talk, Read, Engage, Encourage)** and the **Well Visit Planner** emphasize tailoring guidance to family priorities and daily routines (12, 13). The **Well Visit Planner** gathers pre-visit information that enables clinicians to deliver personalized, goal-oriented support aligned with family strengths and needs (13). Programs like **Talk With Me Baby** extend coaching into everyday caregiving activities such as feeding, diapering, and play, using modeling and reinforcement of warm, responsive, language-rich interactions to build caregiver confidence, strengthen relationships, and foster social-emotional growth (14).

Across these approaches, coaching is operationalized through one or more core actions: (1) education about child development, (2) modeling and demonstration of developmentally supportive behaviors, (4) practice or guided reflection to increase caregiver awareness and responsiveness, (5) collaborative goal setting tailored to family routines and priorities. Together, these approaches suggest that coaching is a key delivery strategy for embedding early relational health into routine pediatric care.

## 5 Discussion

Coaching offers a unifying framework that influences multiple layers of healthcare delivery, shaping clinician behavior, patient and family engagement, and broader system outcomes. In pediatric primary care, coaching extends beyond traditional professional development with the proposed ability to support caregiver behavior change and early relational health. The following sections outline key implications for clinical practice, systems and implementation, and equity and prevention.

### 5.1 Clinical practice implications

Coaching has meaningful implications for clinical practice by reshaping how physicians engage with patients and families. Rather than relying solely on directive guidance, coaching promotes collaborative communication strategies that enhance parent engagement and behavior change. Evidence suggests that while traditional educational approaches can improve clinician knowledge, coaching adds value through individualized feedback, reflection, and goal setting that reinforce sustained changes in practice (15). These approaches align closely with strategies used in health coaching and are particularly relevant for supporting families in adopting developmentally supportive parenting behaviors.

Coaching also strengthens interpersonal and relational skills that are central to effective pediatric care. Through approaches such as peer coaching and reflective feedback, clinicians refine skills in active listening, use of open-ended questions, and strength-based communication (16). These shifts often involves subtle but meaningful changes allowing more space for families to share perspective, slowing the pace of visits, and reducing reliance on jargon, all of which enhance trust and engagement. The effectiveness of these changes is closely tied to the relational dynamic between coach and clinician, where trust and psychological safety promote openness to feedback and sustained growth (1).

Finally, coaching supports practice change within the realities of clinical workflows. Physicians' ability to adopt guideline-based practices is shaped by the stability, resources, and culture of their clinical setting. In environments with limited staffing, high turnover, or resource constraints, coaching often focuses on feasible workflow adjustments rather than large-scale system redesign. Conversely, stable and cohesive practice environments are more conducive to sustained guideline implementation and culture change (1). A meta-analysis found that strategies targeting organizational culture improved physicians' adherence to guidelines, alone or alongside other interventions (15). Coaching is an established tool for building and strengthening team dynamics and organizational culture, which further supports guideline uptake (17). In practice, this may include refining visit structure or workflows, using pre-visit planning tools, training team members to reinforce coaching messages, and identifying which families may need more intensive follow-up. Across contexts, coaching facilitates change at the level of the individual clinician, the care team, and the practice environment (1).

### 5.2 Systems & implementation

Coaching is a powerful tool for supporting Quality Improvement (QI) initiatives, particularly when individual behavior change is aligned with organizational structures, workflows, and culture (1). Peer coaching models, such as the Peer-Coaching for Family Physicians framework (18), help bridge the gap between intention and action by providing clinicians with dedicated time, accountability, and structured support to translate performance data into meaningful practice change (15, 18). Through goal setting, often using framework such as SMART goals (Specific, Measurable, Achievable, Relevant, Time-bound), coaches support clinicians in identifying priorities and implementing incremental, measurable improvements.

In addition to promoting accountability, coaching helps clinicians process feedback and performance data in a constructive way. Without support, audit and feedback processes may evoke feelings of inadequacy or isolation; coaching reframes these experiences as opportunities for growth, strengthening motivation and resilience (18). Central to this process is the creation of psychologically safe learning environments, where coaching is clearly distinguished from evaluation and positioned as formative, developmental support (8).

Beyond its impact on clinicians, coaching also strengthens the broader care relationship. In Health Coaching, family members and friends involved in the patient's care benefit by being part of the supportive system created by the coach, often perceiving the coach's role as supporting the patient between appointments and helping them stay connected to the doctor (4). Studies suggest that patients value the emphases on communication, support, and continuous improvement, and view coaching as a meaningful way to enhance care quality (2). These findings reinforce the feasibility of integrating a coaching stance into routine clinical encounters while maintaining strong patient trust and engagement.

Successful integration of coaching into pediatric primary care will require attention to practical constraints, including limited visit time, competing clinical demands, and variability in team roles. Embedding coaching within existing workflows - such as brief, structured interactions during well-child visits, use of pre-visit tools to identify parent priorities, and reinforcement across longitudinal visits - may enhance feasibility without increasing visit burden. Team-based approaches, including involvement of nurses, medical assistants, or care coordinators, may further support implementation at scale.

### 5.3 Equity and prevention

Coaching has important implications for advancing equity and prevention in pediatric primary care. By focusing on building caregiver capacity, confidence, and self-efficacy, coaching supports families in navigating both developmental challenges and broader social determinants of health. This approach aligns closely with early relational health as a preventive strategy, intervening early to strengthen caregiver-child relationships and reduce the long-term impact of adversity.

Coaching can make developmental guidance more personalized by partnering with families to identify priorities, address barriers, connect with community resources, and incorporate social care into routine pediatric practice. This is particularly important because universal advice may be less effective when families face constraints related to time, stress, language, transportation, or access to supportive services health education or services.

Importantly, early relational health interventions embedded within pediatric care such as those promoting responsive caregiving, early literacy, and parent-child interaction, are strongly linked to long-term developmental, educational, and health outcomes (19). Coaching provides a mechanism for delivering these interventions in a way that is collaborative, culturally responsive, and tailored to family context. Achieving this in practice will require professional development that equips clinicians with coaching competencies and prepares them to adapt recommendations to each family's cultural values, caregiving practices, language preferences, and available resources. Coaching should complement, rather than replace, broader organizational and community efforts to address structural barriers that influence child and family health. In this way, coaching not only supports individual behavior change but also advances a more equitable, relationship-centered model of preventive care.

### 5.4 Limitations

As a conceptual perspective, this manuscript does not present original empirical data. Rather, it synthesizes converging evidence from physician coaching in medical education, health coaching, and parent Despite the shared principles, definitions and implementation approaches vary across the literature, which may limit direct comparisons. In addition, evidence supporting clinician-delivered coaching during routine pediatric primary care remains limited, particularly regarding scalability within time-constrained well-child visits. Future research should evaluate implementation strategies, define standardized coaching competencies and outcome measures, examine effectiveness across diverse pediatric practice settings, and assess potential unintended consequences of applying coaching within caregiver-facing clinical encounters.

### 5.5 Conclusion

Coaching may represent a practical and scalable approach for embedding early relational health within pediatric primary care. By emphasizing collaboration, strengths-based guidance, and shared goal setting, a coaching stance enables clinicians to translate developmental science into strategies that families can integrate into daily routines. Rather than adding a new layer to care, coaching reframes routine well-child interactions to strengthen caregiver confidence and support responsive parent-child relationships. As pediatric primary care continues to expand its role in promoting developmental and behavioral health, coaching offers a feasible and relationship-centered mechanism for integrating early relational health into everyday practice.

## Data Availability

The original contributions presented in the study are included in the article/Supplementary Material, further inquiries can be directed to the corresponding authors.
